# Modification of Extracellular Matrix by the Product of DHA Oxidation Switches Macrophage Adhesion Patterns and Promotes Retention of Macrophages During Chronic Inflammation

**DOI:** 10.3389/fimmu.2022.867082

**Published:** 2022-05-26

**Authors:** Jared L. Casteel, Kasey R. Keever, Christopher L. Ardell, David L. Williams, Detao Gao, Eugene A. Podrez, Tatiana V. Byzova, Valentin P. Yakubenko

**Affiliations:** ^1^ Department of Biomedical Sciences, Quillen College of Medicine, East Tennessee State University, Johnson City, TN, United States; ^2^ Center of Excellence in Inflammation, Infectious Disease and Immunity, Quillen College of Medicine, East Tennessee State University, Johnson City, TN, United States; ^3^ Department of Surgery, Quillen College of Medicine, East Tennessee State University, Johnson City, TN, United States; ^4^ Department of Inflammation and Immunity, Lerner Research Institute, Cleveland Clinic, Cleveland, OH, United States; ^5^ Department of Neurosciences, Lerner Research Institute, Cleveland Clinic, Cleveland, OH, United States

**Keywords:** macrophage, adhesion, migration, integrin α_D_β_2_, CD11d/CD18, carboxyethylpyrrole (CEP), ECM, inflammation

## Abstract

Oxidation of polyunsaturated fatty acids contributes to different aspects of the inflammatory response due to the variety of products generated. Specifically, the oxidation of DHA produces the end-product, carboxyethylpyrrole (CEP), which forms a covalent adduct with proteins *via* an ϵ-amino group of lysines. Previously, we found that CEP formation is dramatically increased in inflamed tissue and CEP-modified albumin and fibrinogen became ligands for α_D_β_2_ (CD11d/CD18) and α_M_β_2_ (CD11b/CD18) integrins. In this study, we evaluated the effect of extracellular matrix (ECM) modification with CEP on the adhesive properties of M1-polarized macrophages, particularly during chronic inflammation. Using digested atherosclerotic lesions and *in vitro* oxidation assays, we demonstrated the ability of ECM proteins to form adducts with CEP, particularly, DHA oxidation leads to the formation of CEP adducts with collagen IV and laminin, but not with collagen I. Using integrin α_D_β_2_-transfected HEK293 cells, WT and $\alpha_{\text{D}}^{-/-}$ mouse M1-polarized macrophages, we revealed that CEP-modified proteins support stronger cell adhesion and spreading when compared with natural ECM ligands such as collagen IV, laminin, and fibrinogen. Integrin α_D_β_2_ is critical for M1 macrophage adhesion to CEP. Based on biolayer interferometry results, the isolated α_D_ I-domain demonstrates markedly higher binding affinity to CEP compared to the “natural” α_D_β_2_ ligand fibrinogen. Finally, the presence of CEP-modified proteins in a 3D fibrin matrix significantly increased M1 macrophage retention. Therefore, CEP modification converts ECM proteins to α_D_β_2_-recognition ligands by changing a positively charged lysine to negatively charged CEP, which increases M1 macrophage adhesion to ECM and promotes macrophage retention during detrimental inflammation, autoimmunity, and chronic inflammation.

## Introduction

Low-grade chronic inflammation is a key component in the development of metabolic and autoimmune diseases such as diabetes, atherosclerosis, obesity, and rheumatoid arthritis (1–3). A critical step in the progression of the disease is the accumulation of classically activated pro-inflammatory (M1-like) macrophages in the extracellular matrix (ECM) of inflamed tissues (4, 5). The failure of pro-inflammatory macrophages to emigrate from the inflamed tissue leads to excessive leukocyte recruitment, metabolic imbalance, and damage of healthy tissue, which all together promote the development of chronic inflammation (1, 6). The mechanism and key components of macrophage retention within the site of inflammation are not yet well understood, although this information can improve the treatment of a variety of different pathologies. Enhanced adhesion that prevents macrophage migration is a potential central mechanism of chronic inflammation development. The cell adhesion force is regulated by the density of adhesive receptors, ligand availability, and receptor-ligand affinity (7). The major adhesive receptors on macrophages are integrins. β_1_ integrins (α_1_β_1_, α_2_β_1_, α_3_β_1_, etc.) have a moderate expression on macrophages (8, 9) and support adhesion/migration to major ECM proteins, such as collagens and laminins. The role of high-density expressed β_2_ integrins (α_M_β_2_, α_D_β_2_, etc.) in macrophage migration/retention in tissue is thought to be limited by lack of binding to major ECM proteins (10, 11).

Nevertheless, we recently demonstrated that macrophage receptor integrin α_D_β_2_ (CD11d/CD18) contributes to the development of atherosclerosis and diabetes due to the generation of strong adhesion of pro-inflammatory macrophages to the inflamed ECM, thereby promoting the retention of macrophages at the site of chronic inflammation (12). Integrin α_D_β_2_ is the most recently discovered leukocyte integrin (13, 14), that contributes to an inflammatory response (12, 15–17) and infection in different pathological conditions (18–21). Interestingly, integrin α_D_β_2_ is upregulated on pro-inflammatory macrophages, which makes it potentially important for macrophage retention (12). However, the critical α_D_β_2_ ligand in the inflamed tissue is not yet defined.

**Figure 1:**
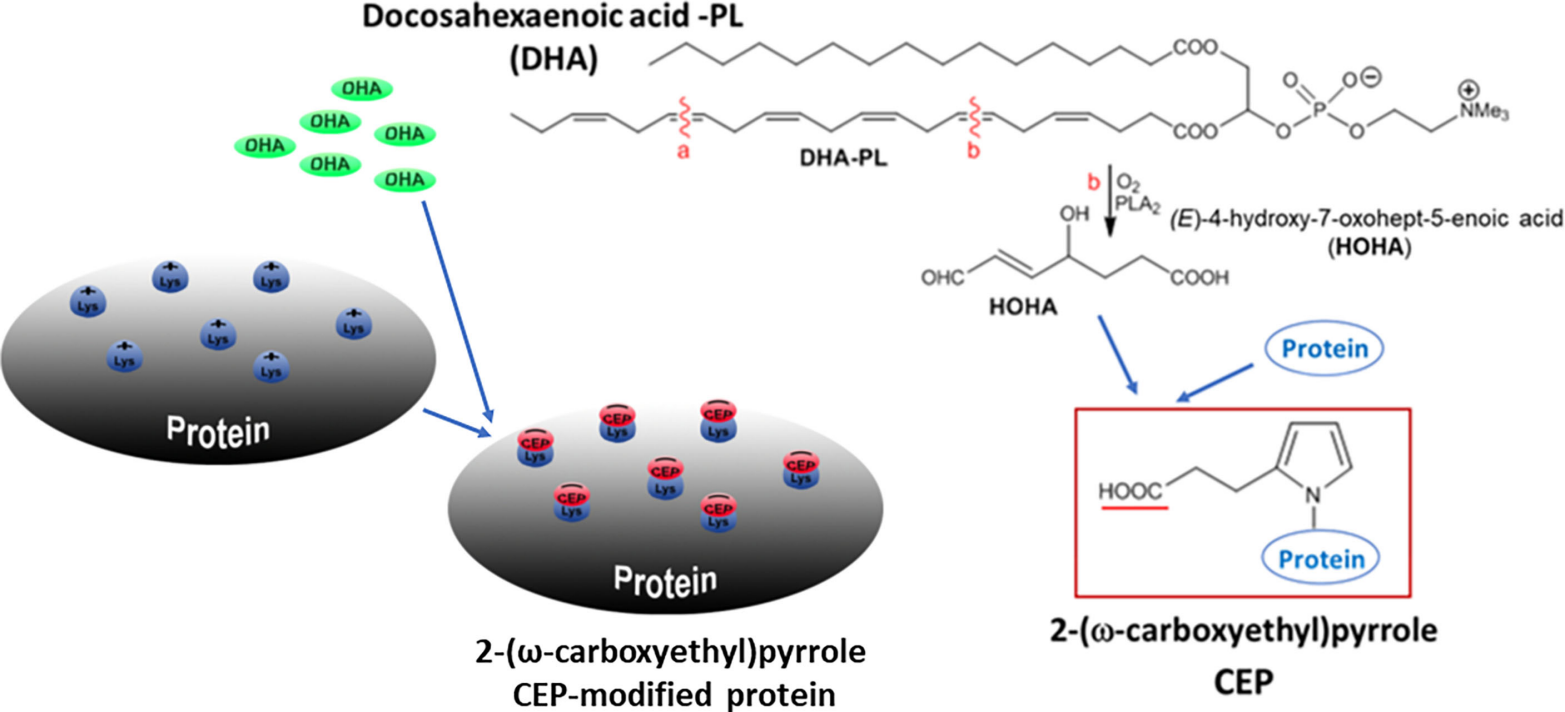
Schematic representation of CEP formation. PLA_2_-catalyzed hydrolysis of DHA generates 4-hydroxy-7-oxo-hept-5-eonate (HOHA), which, in turn, produces 2-ω-Carboxyethylpyrrole (CEP)-protein derivatives through condensation with the primary amino groups of protein lysyl residues. A positively charged lysine is modified by pyrrole with a negatively charged carboxyl group. Multiple lysines can be modified by CEP in one protein molecule.

We recently discovered a potential mechanism that could explain the presence of inflammation-specific ligands in the ECM for integrin α_D_β_2_. We revealed that during acute inflammation, the oxidation of docosahexaenoic acid (DHA) leads to the generation of the end-product carboxyethylpyrrole (CEP), which forms an adduct with proteins *via* an ϵ-amino group of lysines (22, 23). *In vitro* analysis with BSA demonstrated that 10-20 lysines can be modified by CEP in one protein molecule. Notably, positively charged lysines are substituted to the pyrroles with exposed negatively charged carboxyl groups. (**Figure 1**). Based on our recent data, a carboxyl group in the CEP structure is specifically responsible for the binding to integrins α_D_β_2_ and α_M_β_2_ (23). Accordingly, CEP-modification significantly changes the surface charge of the targeted protein and its binding properties.

In our previous study, we found that during peritoneal inflammation at least two proteins are modified with CEP in the peritoneal cavity - fibrinogen and albumin. Most importantly, we found that CEP-modified proteins support the migration of non-polarized macrophages to the site of acute inflammation due to interaction with macrophage integrins α_D_β_2_ and α_M_β_2_ (23). Integrins α_D_β_2_ and α_M_β_2_ are adhesive receptors, which interact with many ligands and transduce a signal to the actin cytoskeleton that regulates cell adhesion, migration, and other cell responses (24, 25). α_D_β_2_ possesses a high homology and similar ligand-binding specificity to α_M_β_2_ (11); however, these two integrins demonstrate different roles in chronic inflammatory diseases. As discussed above, α_D_ supports the development of atherosclerosis (12) and diabetes (26), while integrin α_M_ protects against these metabolic diseases (27, 28). Apparently, the different surface densities of these integrins on primary monocytes (29) and distinct subsets of macrophages (12, 18, 26), determine their diverse roles in cell migration and chronic inflammation. Interestingly, the analysis of α_D_β_2_ and α_M_β_2_ binding to CEP demonstrated that α_D_ has a 5-folds higher affinity to CEP (23), which may have a physiological importance for macrophage adhesion.

The goal of the current project is to evaluate how the modification of ECM proteins with CEP affects their binding properties and the strength of macrophage adhesion, which is critical for macrophage retention during chronic inflammation.

## Material and Methods

### Reagents and Antibodies

Reagents were purchased from Sigma-Aldrich (St. Louis, MO) and Thermo Fisher Scientific (Waltham, MA). Recombinant mouse IFNγ, CCL2, and CCL5 were purchased from Invitrogen Corporation (Carlsbad, CA). Human fibrinogen and thrombin were obtained from Enzyme Research Laboratories (South Bend, IN). Collagen IV, collagen I, and laminin were purchased from Corning (Corning, NY). CEP-BSA (21:1 molar ratio) and CEP-Fg (29:1 molar ratio) were prepared through a Paar-Knorr pyrrole synthesis with (9H-fluoren-9-ylmethyl ester 4,7-dioxo-heptanoic acid) DOHA-Fm as described previously (28). Anti-CD68 mAb was from eBioscience. Polyclonal antibody against the α_D_ I-domain was described previously (11). The antibody recognizes both human and mouse α_D_ I-domains and has no cross-reactivity with recombinant human and mouse α_M_, α_X,_ and α_L_ I-domains. The antibody was isolated from rabbit serum by affinity chromatography using α_D_ I-domain-Sepharose. Anti-macrophage antibody F4/80 was from eBioscience (San Diego, CA). A polyclonal antibody against CEP and monoclonal IgM anti-CEP antibody were described previously (30, 31). Blocking IgG anti-CEP antibody (Clone 3C9) was generated in Dr. Tatiana Byzova’s laboratory (23).

### Animals

Wild type (C57BL/6J, stock # 000664) and integrin α_D_-deficient (B6.129S7-*Itgad^tm1Bll^*/J, stock # 005258) mice were bought from Jackson Laboratory (Bar Harbor, ME). α_D_-deficient have been backcrossed to C57BL/6 for at least ten generations. All procedures were performed according to animal protocols approved by East Tennessee State University IACUC.

### Generation of Classically Activated (M1) Macrophages

Notably, the terms M1 and M2 macrophages are artificial and do not represent all variabilities of macrophage subsets *in vivo*. We used the term M1 in our manuscript to refer to *in vitro* polarized macrophages activated by IFN-γ which serve as a model for pro-inflammatory macrophages *in vivo*. Therefore, peritoneal macrophages from 8-12-week-old mice (WT and $\alpha_{\text{D}}^{-/-}$ ) were harvested by lavage of the peritoneal cavity with 5 ml of sterile PBS 3 days after intraperitoneal (IP) injection of 4% thioglycollate (TG; 0.5ml). The cells were washed twice with PBS and resuspended in RPMI media supplemented with 10% FBS, 0.1 mg/ml streptomycin, and 0.1 unit/ml penicillin. The cell suspension was transferred into 100mm Petri dishes and incubated for 2h at 37°C in humidified air containing a 5% CO_2_ atmosphere. Nonadherent cells were washed out with PBS, and the adherent macrophages were replenished with complete RPMI media. The macrophages were differentiated to M1 phenotypes by treatment with recombinant mouse interferon-γ (IFN-γ) (100 U/ml, Thermo Fisher) for 4 days. Medium with IFN-γ was changed every 2 days or as required. Macrophages were dissociated from the plates using 5mM EDTA in PBS and used for the experiments thereafter.

### Generation of α_D_β_2_-Transfected HEK293 Cells

The HEK293 cells stably expressing human integrin α_D_β_2_ were generated as described previously (11) and maintained in DMEM/F-12 (Invitrogen) supplemented with 10% FBS, 2 mM glutamine, 15 mM HEPES, 0.1 mg/ml streptomycin, and 0.1 unit/ml penicillin.

### Isolation of Recombinant α_D_ I-Domain in an Active Conformation

The construct for the α_D_ I-domain was generated and recombinant protein was isolated as described in our previous paper (23). Briefly, α_D_ in the active conformation (Pro^128^-Lys^314^) was inserted in the pET15b vector, expressed in E. Coli as a His-tag fusion protein, and purified using affinity chromatography on Ni-chelating agarose (Qiagen Inc., Valencia, CA).

### Flow Cytometry Analysis

Flow cytometry analysis was performed to assess the expression of integrin α_D_ on mouse peritoneal macrophages and α_D_β_2_-transfected HEK293 cells. Cells were harvested and pre-incubated with 4% normal goat serum for 30 min at 4°C, then 2x10^6^ cells were incubated with a specific antibody for 30 min at 4°C. Non-conjugated antibodies required additional incubation with Alexa 488 or Alexa 647-donkey anti-mouse IgG (at a 1:1000 dilution) for 30 min at 4°C. Finally, the cells were washed and analyzed using a Fortessa X-20 (Becton Dickinson).

### Cell Adhesion Assay

The adhesion assay was performed as described previously (23) with modifications. Briefly, 96-well plates (Immulon 2HB, Cambridge, MA) were coated with different concentrations of fibrinogen, collagen IV, collagen I, or laminin for 3 h at 37°C. The wells were post-coated with 0.5% polyvinyl alcohol for 1 h at room temperature. Mouse peritoneal macrophages or HEK293 cells transfected with integrin α_D_β_2_ were labeled with 10 µM Calcein AM (Molecular Probes, Eugene, OR) for 30 min at 37°C and washed with HBSS and resuspended in the same medium at a concentration of 1 × 10^6^ cells/mL. α_D_β_2_-transfected HEK 293 cells were also supplemented with 2mM MnCl_2_. Aliquots (50 µL) of the labeled cells were added to each well. After 30 minutes of incubation at 37°C in a 5% CO_2_ humidified atmosphere, the nonadherent cells were removed by washing with PBS. The fluorescence was measured in a Synergy H1 fluorescence plate reader (BioTek, Winooski, VT), and the number of adherent cells was determined from a labeled control.

### Cell Spreading Assay

Glass coverslips were coated with fibrinogen (20 µg/ml), Collagen IV (5 μg/ml) or CEP-BSA (750 μM) for 3 hours at 37°C and post-coated with 0.5% polyvinylpyrrolidone for 1 hour at 37°C. To determine cell spreading, inflammatory macrophages isolated from the WT and $\alpha_{\text{D}}^{-/-}$ mice were allowed to adhere to coverslips for 1 hour at 37°C. Macrophages were fixed with 4% paraformaldehyde, permeabilized with 0.1% Triton X-100, and incubated with Phalloidin-iFluor 555 and DAPI. Fluorescent images were obtained with EVOS FL auto cell fluorescent imaging system using 20x objective with multi-frame automatic setup (9-fields). The cell areas were calculated using Imaris 8.0 software.

### Migration of Macrophages in 3D Fibrin Gel

Migration assay was performed as described previously (26). Polarized M1 macrophages were labeled with PKH26 red fluorescent dye. Cell migration assay was performed for 48 hours at 37°C in 5% CO_2_ in a sterile condition. An equal number of WT macrophages was evaluated by cytospin of mixed cells before the experiment and at the starting point before migration. Labeled WT (1.5x10^5^) activated macrophages were plated on the membranes of transwell inserts with a pore size of 8 μm and 6.5 mm in diameter (Costar, Corning, NY) precoated with fibrinogen (Fg). Fibrin gel (100 µl/sample) was generated by mixing 0.75mg/ml Fg containing 1% FBS and 1% P/S and 0.5 U/ml thrombin. In some samples 9 µM CEP was incorporated into the gel during polymerization. 30 nM of MCP-1 was added on top of the gel to initiate the migration. Migrating cells were detected by Leica Confocal microscope (Leica-TCS SP8), and the results were analyzed and reconstructed using IMARIS 8.0 software.

### Biolayer Interferometry

The binding parameters of the interaction between the α_D_ I-domain with CEP or fibrinogen were determined using an Octet K2 instrument (FortéBio, Sartorius Group). CEP-BSA or fibrinogen was immobilized on the Amine Reactive Second-generation (AR2G) biosensor using the amine coupling kit according to the manufacturer’s protocol. Different concentrations of the active forms of α_D_ I-domain (50-1000 nM) were applied in the mobile phase, and the association between the immobilized and flowing proteins was detected. Experiments were performed in 20 mm HEPES, 150 mm NaCl, 1 mM CaCl_2_, 1 mM MgCl_2_, 0.02% (v/v) Tween-20, 0.02% BSA, pH 7.5. The protein-coated biosensors were regenerated with 25 mm NaOH and 2M NaCl. Analyses of the binding kinetics were performed using DataAnalysis HT11.0 software (ForteBio). The value of the equilibrium dissociation constant (K_D_) was obtained by fitting a plot of response at equilibrium (Req) against the concentration.

### Immunostaining

Cryosections (10 μm) of aorta roots (from WT and ApoE^-/-^ mice after five weeks on a western diet) were warmed to room temperature for 30 minutes prior to immunofluorescence staining. Tissue sections were fixed in ice-cold acetone for 10 minutes followed by permeabilizing with 0.2% Tween-20 for 10 min to increase the signal of intracellular binding sites. Tissue sections were washed in PBS and incubated with SuperBlock (PBS) Blocking buffer (Thermo Scientific, Rockford, IL, USA) for 45 min to block nonspecific binding. Tissues were then incubated at 4°C overnight with the primary antibody (rabbit polyclonal anti-CEP and rat anti-mouse CD68 (macrophage marker). After washing several times with PBS, the sections were incubated with Alexa Fluor 488-conjugated donkey anti-rabbit IgG and Alexa Fluor 568-conjugated donkey anti-rat for 1 hour at room temperature. The sections were subsequently washed and sealed. The tissue sections were examined by Leica Confocal microscope (Leica-TCS SP8). Control sections without the primary antibody were also performed at the same time.

### Analysis of CEP Formation in Atherosclerotic Lesions of ApoE^-/-^ Mice by Western Blot

Male ApoE-deficient mice were kept on a Western diet for 16 weeks. After that, ApoE^-/-^ and the same age WT C57BL/6 mice (as the control) were euthanized, their vascular system was perfused with 10 ml PBS, and the aortas were cleaned from the outer layers of connective tissue and removed. The isolated aortas were digested with Collagenase type I, type XI, Hyaluronidase, and DHAase I according to the published protocol (12). The cells were separated by centrifugation. The non-cellular supernatant was high speed centrifugated to exclude aggregates and cell traces, and used for Western Blot analysis.

The same concentration of proteins isolated from ApoE^-/-^ and WT aortas was loaded on SDS-electrophoresis analyzed by 7% SDS-PAGE electrophoresis and Western blotting. The Immobilon-P membranes (Millipore) were incubated with rabbit polyclonal anti-CEP antibody, followed by incubation with goat anti-rabbit secondary antibody conjugated to horseradish peroxidase and developed using enhanced SuperSignal Chemiluminescent Substrate (Pierce).

### Generation of CEP-Modified ECM Proteins

The evaluation of CEP-modified proteins was performed as described previously (23). Collagen I, collagen IV, and laminin were coated on a 96-well plate at a concentration of 20 μg/ml overnight at 4°C. Wells were post-coated with 0.5% polyvinyl alcohol for 1 h at 37°C. 20 μM DHA in 20mM Hepes, 150 mM NaCl, 0.01% H_2_O_2_, and 1mM CaCl_2_ were added to the wells and incubated in an oxygen-free environment (under argon) for 18 hours at 37°C. After incubation, the plate was washed out with PBS supplemented with 0.05% Tween-20 3 times and incubated with anti-CEP polyclonal antibody (0.9 μg/ml) for 2 hours at 37°C. After washing, wells were incubated with goat-anti-rabbit HRP conjugated antibody for 1 h at 37°C and the binding was developed using TMB-ELISA substrate solution (Pierce). The result was detected by a plate reader using a wavelength of 450 nm.

### Statistical Analysis

Statistical analyses were performed using a Student’s t-test or Student’s paired t-tests where indicated in the text using SigmaPlot 13. A value of p<0.05 was considered significant. Error bars represent the SEM of experiments.

## Results

### DHA Oxidation Generates CEP Adducts With ECM Proteins in Atherosclerotic Lesions and *In Vitro*

**Figure 2:**
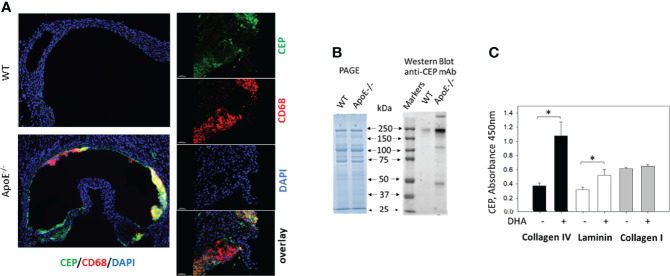
CEP forms adducts with ECM proteins during inflammation and oxidation of DHA. **(A)** CEP deposition in atherosclerotic lesions. Aortic sinuses were isolated from the ApoE-deficient mice after five weeks on a western diet. The sections were prepared and immunostained with anti-CEP (green) and anti-CD68 (red) antibodies. The crosssection of entire aorta roots (right) and 200x magnified inlet (left) are shown (scale bar 200 μm). **(B)** Electrophoresis (PAGE) and Western blot analysis with anti-CEP antibody of digested aortas. Aortas of ApoE-/- and WT mice were digested, cells were separated and the non-cellular contest was analyzed by electrophoresis with Coomassie Blue and Western Blot (7% PAGE) with anti-CEP antibody (representative experiment from 4 performed). **(C)** Generation of CEP-modified ECM proteins. Collagen IV, collagen I, and laminin were coated on a 96-well plate and incubated in the presence (or absence) of 2 μM DHA in 20 mM HEPES, 150 mM NaCl, 0.01% H_2_O_2_, and 1mM CaCl_2_ in an oxygen-free environment (under argon atmosphere) for 18 hours at 37°C. After incubation, the plate was incubated with an anti-CEP polyclonal antibody (0.9 μg/ml) followed by goat-anti-rabbit HRP conjugated antibody. The binding was developed using a TMB-ELISA substrate solution (Pierce). The result was detected by plate reader using wavelength 450 nm. Statistical analyses were performed using Student’s paired *t*-tests from 3 independent experiments. *P < 0.05.

We and others have demonstrated the presence of CEP in different inflammatory tissues using immunohistochemistry (23, 30–34). Particularly, anti-CEP antibody detected strong immunostaining in the sections of atherosclerotic lesions of ApoE^-/-^ mice on a Western diet (**Figure 2A**). However, the direct formation of CEP-protein adducts was shown previously only in the peritoneal cavity, but not in the tissue (23). To demonstrate the link between CEP immunostaining and the formation of CEP-modified proteins in the inflamed tissue during chronic inflammation, we evaluated CEP-protein adduct formation in atherosclerotic lesions (**Figure 2B**). Male ApoE-deficient mice were kept on a Western diet for 16 weeks (n=3). After that, ApoE^-/-^ and the same age WT C57BL/6 mice (as the control) were euthanized, their vascular system was perfused and aortas were removed. The isolated aortas were digested according to the published protocol (12) and cells were separated by centrifugation. The non-cellular supernatant was high speed centrifugated to exclude aggregates and cell traces, and used for Western Blot analysis.

The same concentration of proteins isolated from ApoE^-/-^ and WT aortas was loaded on SDS-electrophoresis (**Figure 2B**, left panel) and analyzed using an anti-CEP antibody (**Figure 2B**, right panel). We found a dramatic difference in anti-CEP staining of proteins isolated from the atherosclerotic lesions of ApoE-deficient and WT mice. While only one weak band was spotted in the sample isolated from WT mice, several intensive bands were detected in the ApoE^-/-^ mice samples that represent several CEP-modified proteins. Notably, the faded CEP signal from the WT mice corresponds to the concept that the natural oxidation of PUFA in a healthy organism can lead to a low level of adduct formation. We detected such signal previously in commercial fibrinogen that we used in our experiments as a control (23).

To confirm the ability of CEP to form the covalent adduct with ECM proteins, we tested the formation of CEP modification in collagen I, laminin, and collagen IV using *in vitro* DHA oxidation assay that we developed previously (23). ECM proteins were incubated with DHA and 0.01% H_2_O_2_ for 18 h at 37°C. After incubation, CEP formation was detected by ELISA with an anti-CEP antibody (**Figure 2C**). We found that DHA oxidation led to the formation of CEP-collagen IV adduct and CEP-laminin adduct, while collagen I was not modified.

These experiments clearly demonstrated that ECM proteins are modified by CEP, and inflamed tissue (e.g. atherosclerotic lesions) can be a source of specific adhesive substrate for macrophages.

### M1 Macrophages Demonstrate Stronger Adhesion and Spreading to CEP-Modified Proteins

**Figure 3:**
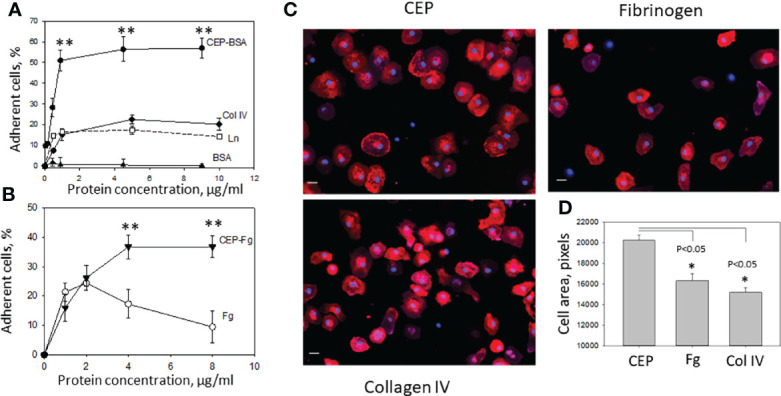
Adhesion of M1-polarized macrophages to CEP and ECM proteins. **(A)** Different concentrations of CEP-BSA, collagen IV (Col IV), laminin (Ln) and BSA were immobilized on a 96-well plate for 3 h at 37°C. Fluorescently labeled macrophages were added to the wells and cell adhesion was determined after 30 min in a fluorescence plate reader. CEP-BSA is presented based on the concentration of BSA. 1µg BSA contains 250 nM CEP. A representative experiment from 4 performed. Data are presented as mean ± SEM **p < 0.01. **(B)** Different concentrations of CEP-fibrinogen (CEP-Fg) and fibrinogen (Fg) were immobilized and tested in an adhesion assay as described above. A summary result from 3 experiments. CEP-Fg is shown based on the concentration of Fg. 1µg Fg contains 87 nM CEP Data are presented as mean ± SEM **p < 0.01. **(C)** Macrophages were allowed to adhere to glass coverslips coated with fibrinogen, Collagen IV or CEP-BSA for 1 hour at 37°C. Macrophages were fixed with 2% paraformaldehyde, permeabilized with 0.1% Triton X-100, and incubated with Alexa Fluor 555-conjugated phalloidin and DAPI. Fluorescent images were obtained with EVOS FL auto cell fluorescent imaging system using 20x objective with mosaic 9-fields setup. **(D)** The cell area was calculated using Imaris 8.0 software. Data are presented as mean ± SEM *p < 0.05.

Pro-inflammatory, M1-like macrophages, are the major source of inflammatory stimuli during chronic inflammation. We tested how protein modification with CEP affects M1 macrophage adhesion to ECM proteins such as collagen IV, laminin, and fibrinogen (which leaks from blood to the ECM through the damaged endothelial monolayer during inflammation). Different concentrations of collagen IV, laminin, CEP-modified BSA, and BSA alone as a control were immobilized on the 96-well plate and tested as substrates for M1 macrophage adhesion (**Figure 3A**). We found that the adhesion of pro-inflammatory macrophages to CEP-modified BSA dramatically overreached the adhesion of macrophages to natural ECM ligands. Notably, BSA alone does not support the adhesion of macrophages. In a separate experiment, we compared the adhesion of fibrinogen-modified by CEP and native fibrinogen. Clearly, CEP modification dramatically increased macrophage adhesion to fibrinogen (**Figure 3B**).

To verify these results macrophage spreading on CEP, collagen IV, and fibrinogen was evaluated. Cells were labeled with phalloidin-PE, examined by fluorescent microscopy, and analyzed using the ImarisCell module (Imaris 8.0). Corresponding to the adhesion data, the results demonstrate stronger spreading to CEP with larger cell areas and focal adhesion complexes (**Figures 3C, D**).

### The Presence of CEP in a 3D Matrix Inhibits M1 Macrophage Migration

**Figure 4:**
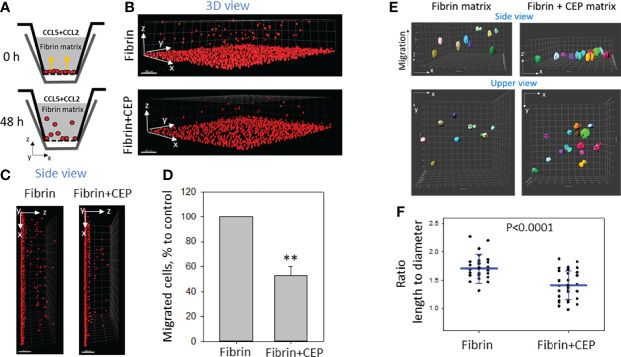
The presence of CEP in the matrix inhibits the 3D migration of M1 macrophages. **(A)** Sketch diagram of the migrating cells in Boyden transwell chamber. Before migration (upper panel) and after 48h migration (lower panel). **(B)** WT M1 macrophages were labeled with red PKH26 fluorescent dye. Labeled WT (1.5x10^5^) activated macrophages were plated on the membranes of transwell inserts (Boyden chamber) with a pore size of 8 μm and 6.5 mm in diameter (Costar, Corning, NY) precoated with fibrinogen (Fg). Fibrin gel (100 µl/sample) was generated by mixing 0.75mg/ml Fg containing 1% FBS and 1% P/S and 0.5 U/ml thrombin with or without 9uM CEP. Migration of macrophages was stimulated by 30 nM CCL2 and 100nM CCL5 added to the top of the gel. After 48 hours, migrating cells were detected by a Leica Confocal microscope **(B, C)**. **(D)** The results were analyzed by IMARIS 8.0 software, and statistical analyses were performed using Student’s paired t-tests (n = 5 per group). Scale bar= 300 μm. Data are presented as mean ± SEM. **P < 0.01. **(E)** The morphology of non-labeled macrophages was evaluated after gel staining with CytoPainet Phalloidinin-iFluor 555 in fibrin matrix (left panel) and Fibrin matrix supplemented with CEP (right panel). The ratio length/diameter was calculated using the ImarisCell module (IMARIS 8.0). **(F)** Statistical analyses were performed using Student’s t-tests, n = 30 (fibrin gel) 36 (fibrin+CEP gel). P < 0.0001.

To evaluate the effect of CEP modification in ECM on cell motility we performed a 3D migration assay by comparing M1 macrophage migration in a polymerized fibrin matrix and polymerized fibrin matrix supplemented with 9 μM CEP. Based on our previous result the migration of M1 macrophages in a fibrin matrix is lower compared to non-activated or M2 macrophages (26). The goal of this experiment was to evaluate the additional effect of CEP on this migration. Cells were plated on one side of the gel and migration was initiated by CCL2 and CCL5 added to the other side of the gel (**Figure 4A**). We found that migration of M1 macrophages was dramatically reduced in fibrin gel supplemented with CEP (**Figures 4B–D**), which corresponds to strong adhesion events and promotion of macrophage retention at the site of chronic inflammation.

To more critically evaluate these results, we tested the morphology of macrophages in a 3D matrix supplemented with CEP. 3D gels were generated using polymerized fibrin and polymerized fibrin/9 uM CEP. The same macrophages were incorporated into both matrixes during polymerization and migration was stimulated with a gradient of CCL2. After 18 hours, the cells inside the gel (at least 80 µM from the gel surface) were analyzed using the confocal microscope. We found that cells in the fibrin matrix present a more elongated shape when compared with the more rounded cells in the matrix with supplemented CEP (**Figure 4E**) The difference between the length-to-diameter ratio was extremely significant (P<0.0001, n=30-35/group) (**Figure 4F**). According to published data, elongated cell morphology in a 3D matrix corresponds to migrating cells.

Therefore, our results demonstrate that modification with CEP affects M1 macrophage adhesion, spreading, and migration-dependent morphology.

### Integrin α_D_β_2_ Is Critical for M1 Macrophage Adhesion and Spreading to CEP-Modified Proteins

**Figure 5:**
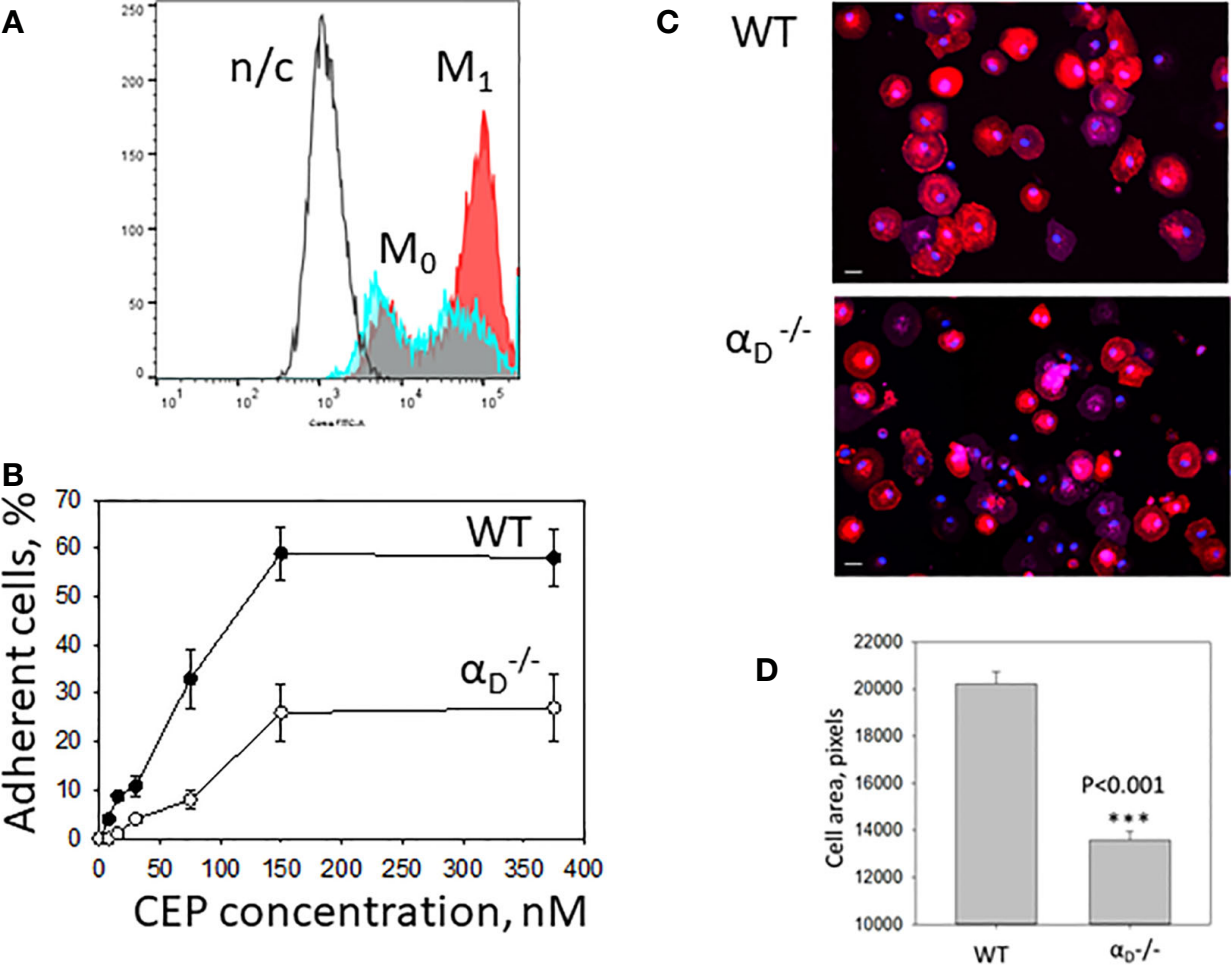
Integrin α_D_β_2_ is critical for M1 macrophage adhesion to CEP-modified proteins. **(A)** The expression of integrin α_D_ is upregulated on M1 macrophages. Peritoneal macrophages were isolated and plated for 4 days in the presence of IFNg in complete RPMI media (Red). M1 macrophages were collected using cell dissociation buffer and stain with anti-α_D_ polyclonal antibody, followed by staining with donkey-anti-rabbit Alexa 647secondary antibody. Freshly isolated peritoneal macrophages were used as a control (M0)(Blue). Integrin α_D_ expression was tested using flow cytometry (Fortessa X-20). N/c-negative control. **(B)** Adhesion of WT and $\alpha_{\text{D}}^{-/-}$ M1 macrophages to CEP-BSA. Isolated M1 macrophages were fluorescently labeled with Calcein AM and added to the well coated with CEP-BSA. cell adhesion was determined after 30 min in a fluorescence plate reader. **(C)** Macrophages were allowed to adhere to glass coverslips coated with CEP-BSA for 1 hour at 37°C. After fixation with 2% paraformaldehyde and permeabilization with 0.1% Triton X-100, cells were incubated with CytoPainet Phalloidinin-iFluor 555 and DAPI. Fluorescent images were obtained with EVOS FL auto cell fluorescent imaging system using 20x objective with mosaic 9-fields setup. **(D)** The cell area was calculated using Imaris 8.0 software. Data are presented as mean ± SEM ***p < 0.001.

The major adhesive receptors for CEP on macrophages are integrins α_D_β_2_ and α_M_β_2_. Integrin α_D_ is upregulated on M1 macrophages (12), (**Figure 5A**), while α_M_ maintains the same level of expression. This difference determines the distinct roles of these β_2_ integrins in inflammation. While α_M_β_2_ supports macrophage migration and demonstrates some protective function during chronic inflammation (27, 35), integrin α_D_β_2_ inhibits M1 macrophage migration and contributes to the development of chronic inflammation (12, 26). We hypothesized that integrin α_D_β_2_ is critical for M1 macrophage adhesion to CEP-modified proteins. To test this hypothesis, we compared the adhesion of M1 macrophages isolated from the WT and α_D_-deficient mice to CEP. The adhesion assay demonstrated a dramatic reduction in macrophage adhesion in α_D_-deficient mice (**Figure 5B**). Moreover, macrophage spreading to CEP was significantly affected in $\alpha_{\text{D}}^{-/-}$ M1 macrophages, the total cell areas were reduced as well as cell shapes were irregular (**Figures 5C, D**).

Therefore, our data demonstrate that α_D_-deficiency significantly reduced adhesion and spreading of M1 macrophages on CEP, which confirmed the critical role of α_D_β_2_ in macrophage adhesion and potential retention in CEP-modified substrate.

### The Binding Properties of CEP Exceed the Binding Properties of Natural α_D_β_2_ Ligands

**Figure 6:**
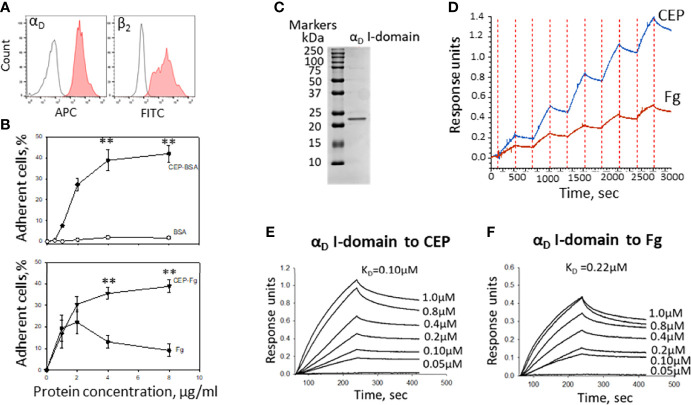
The binding properties of CEP exceed the binding properties of natural α_D_β_2_ ligands. **(A)** The expression of integrins α_D_ and β_2_ on the surface of HEK293-transfected cells. After isolation, cells were stained with anti-α_D_ polyclonal antibody and anti-β_2_ monoclonal antibody, followed by staining with donkey-anti-rabbit Alexa 647 and donkey-anti-mouse Alexa 488 secondary antibodies. Control is shown in open histogram. **(B)** Different concentrations of CEP-BSA and BSA (Upper panel) or CEP-fibrinogen (CEP-Fg) and Fg (lower panel) were immobilized on a 96-well plate for 3 h at 37°C. Fluorescently labeled α_D_β_2_ HEK293-transfected cells were added to the wells and cell adhesion was determined after 30 min in a fluorescence plate reader. A summary result from 4-6 experiments. Data are presented as mean ± SEM **p < 0.01. **(C)** Isolated α_D_ I-domain binds to CEP in biolayer interferometry assays. SDS-PAGE of generated I-domain. α_D_ active (D^122^-K^314^) I-domain was isolated from soluble fractions of **(E)** coli lysates and purified using affinity chromatography. The protein purity was assessed by SDS-PAGE on 4-15% gradient gel under non-reducing conditions followed by staining with Coomassie Blue. Representative profiles of the biolayer interferometry responses for α_D_ binding to the immobilized CEP-BSA and Fibrinogen (Fg). **(D)** Single cycle kinetic of α_D_ I-domain binding (concentrations ranging from 24-750 nM) to CEP-BSA and Fg coupled to AR2G biosensor. **(F)** Steady state analysis of α_D_ I-domain binding to CEP **(E)** and Fg **(F)** (concentrations ranging from 50-1000 nM). The K_D_ of binding was calculated using DataAnalysis HT11.0 software (ForteBio).

Macrophages are a complex system with a variety of adhesive receptors that have overlapping ligand binding properties. To verify the role of CEP as a strong α_D_β_2_ ligand, we tested α_D_β_2_-transfected HEK293 cells (**Figure 6A**). HEK293 cells do not express any receptors that interact with CEP such as integrin α_M_β_2_, CD36, or TLRs (23, 34). Adhesion of α_D_β_2_-transfected HEK293 cells was tested to CEP-BSA versus BSA and CEP-fibrinogen versus fibrinogen. As we expected α_D_β_2_-cells do not adhere to BSA, but demonstrate the strong concentration-dependent adhesion to CEP-BSA. (**Figure 6B**, upper).

Fibrinogen is a natural ligand for integrin α_D_β_2_ and a common element of the ECM during inflammation. Markedly, CEP modification significantly improves the adhesive properties of fibrinogen to α_D_β_2_-HEK293 transfected cells similar to the result, which was obtained for M1 macrophages (**Figure 3B**). Notably, it has been shown previously that cell adhesion to fibrinogen achieved maximum at fibrinogen concentration 2-3 µg/ml and is significantly reduced at higher concentrations (10, 36). The mechanism is not known but may depend on the surface-induced lateral aggregation of fibrinogen (37). This result demonstrated that CEP modification in different proteins provides a similar augmentation of cell adhesive properties.

To evaluate a difference in the binding of α_D_β_2_ to CEP and fibrinogen, we isolated α_D_β_2_ ligand-binding motif – I-domain (**Figure 6C**) and examined its binding properties using BioLayer Interferometry. CEP and fibrinogen were immobilized on amino coupling biosensors and tested with different concentrations of α_D_ I-domain using two approaches such as kinetic titration (single cycle kinetics) (**Figure 6D**) and equilibrium analysis (steady-state analysis) (**Figures 6E, F**). The binding affinity was analyzed using DataAnalysis HT11.0 software (ForteBio). We found that the affinity of α_D_ binding to CEP (K_D_=1.0x10^-7^M) is 2-fold stronger than the α_D_ interaction with fibrinogen (K_D_=2.2x10^-7^).

Therefore, α_D_β_2_ generates a more powerful adhesion to CEP-modified proteins due to a higher affinity mediated by a negative charge of a carboxyl group in CEP’s structure. This result suggests the unique role of CEP-modified ECM proteins in macrophage adhesion that generates strong α_D_β_2_-dependent adhesion *via* CEP that can be critical for macrophage retention.

## Discussion

In our previous project, we found that CEP, the product of DHA oxidation, is a ligand for integrins α_M_β_2_ and α_D_β_2_ (23). In the current study, we tested how the modification of the ECM proteins with CEP affects macrophage adhesion. Now we found that 1) Polyunsaturated fatty acid oxidation generates the formation of adducts between CEP and ECM proteins *in vitro*. 2) The development of atherosclerosis promotes a CEP formation in proteins within the atherosclerotic lesions. 3) Pro-inflammatory M1 macrophages demonstrate a stronger adhesion to CEP-modified proteins compared to natural ECM ligands. 4) M1 macrophage adhesion to CEP inhibits cell migration. 5) The adhesion of M1 macrophages to CEP-modified proteins is driven by integrin α_D_β_2_.

The previous experiments using immunostaining with the anti-CEP antibody demonstrated the deposition of CEP in tissue during inflammation including myocardial infarction (38), peritoneal inflammation (23), atherosclerosis (38), and macular degeneration (32, 39). Our published data revealed that neutrophil-mediated oxidation led to the formation of CEP-fibrinogen and CEP-BSA adducts (23). We hypothesize that PUFA oxidation can lead to the modification of different ECM proteins that contain an exposed lysine on a protein’s surface. We do expect some limitations, which can depend on special tertiary structures, post-translational modifications, or cross-linkage through lysines. Our concern was primarily based on the absence of CEP adduct formation with the E-fragment of fibrin (23), which has a strong triple-coiled helix structure. (The triple-coiled helix is also a major component of laminin and collagen fibrils). In addition to a triple-coiled structure, collagens IV, V, and VI contain flexible globular domains, which are absent in collagens I and III (40). In our current experiments, we found that *in vitro* oxidation of DHA generates CEP-collagen IV adduct formation, while collagen I is not modified by CEP, which confirms our hypothesis regarding the potential role of the globular domains in CEP adducts formation.

Previously we demonstrated that non-activated peritoneal macrophages can bind CEP *via* α_M_β_2_ and α_D_β_2_. However, we did not compare the level of macrophage adhesion to natural adhesive ligands and CEP-modified ligands. Moreover, we did not test the effect of CEP modification on the adhesion and migration of M1 macrophages, which is critical for the development of inflammatory response. In this paper, we are particularly focused on integrin α_D_β_2_ since it is upregulated on pro-inflammatory M1-like macrophages and our previous data demonstrated that the α_D_ I-domain showed 5 folds stronger affinity to CEP compared to the α_M_ I domain (23, 41). This difference most likely is mediated by clusters of positively charged amino acids on the surface of the α_D_ I-domain. The increased binding activity to negatively charged ligands was described previously for integrin α_X_β_2_ which has a significantly lower expression on macrophages but has a high level of homology with integrin α_D_β_2_ (42).

In our previous project, we found that α_D_β_2_ on the surface of pro-inflammatory M1 macrophages contributed to the retention of macrophages at the site of atherosclerotic lesions due to strong cell adhesion (12). It has been shown that α_D_β_2_ is a multiligand protein that can bind to ICAM-3, VCAM-1, fibrinogen, fibronectin, and vitronectin (11). However, these proteins are cell surface receptors or plasma proteins, which are sporadic in the interstitium, while the major ECM proteins such as collagens and laminins do not support α_D_β_2_ -mediated adhesion (26). This raises the question regarding the potential ECM ligands for α_D_β_2_ -mediated macrophage retention. The modification of collagen IV and laminin with CEP (**Figure 2C**) demonstrates the protein modification in ECM and resolves this issue. This mechanism presents the formation of the universal ligand for α_D_β_2_-mediated adhesion. Notably, in our previous project, we found that a carboxyl group in CEP’s structure is critical for integrin binding since another product of DHA oxidation, ethylpyrole, has a similar structure, but does not have a carboxyl group and loses the ability to interact with integrins α_M_β_2_ and α_D_β_2_ (23). Therefore, the positively charged lysines in different proteins can be modified with negatively charged CEP, which converts these proteins to the α_D_β_2_-dependent substrate.

We found that adhesion and spreading of M1 macrophages to the CEP-modified proteins is much stronger compared to native collagen IV, laminin, or fibrinogen (**Figure 3**). The adhesion to collagens and laminins is mediated *via* β_1_ integrins such as α_1_β_1_, α_2_β_2_, etc. The level of β_1_ integrins is lower on macrophages compared to β_2_ integrins (8, 9). Therefore, the switch of binding from classical collagen/β_1_-dependent to CEP-modified collagen/β_2_-dependent increased cell adhesion. Moreover, we demonstrated that M1 macrophage adhesion to CEP is integrin α_D_β_2_-dependent since it is dramatically reduced in α_D_-deficient macrophages (**Figure 5B**). Notably, α_D_-deficiency does not affect the expression of other major adhesive receptors on the macrophage surface (26). We verified this result on several systems including α_D_β_2_-transfected cells and isolated α_D_ I-domain. α_D_β_2_-transfected cells generated lower adhesion compared to M1 macrophages (**Figures 5B**, **6B**) since the expression of α_D_β_2_ on these cells is lower (**Figures 5A**, **6A**). Nevertheless, these cells demonstrate a specific effect of integrin α_D_β_2_ on cell adhesion. The results of the adhesion assay are confirmed by BioLayer Interferometry, which measures the direct protein-protein interaction between α_D_ I-domain and CEP or fibrinogen.

I-domain is a region of α_D_β_2_ that contains most of the ligand-binding sites for β_2_ integrins (43). We previously showed that binding sites for CEP and fibrinogen are located within the I-domain (23). In our earlier study regarding the ligand binding properties of integrin α_D_β_2,_ we compared α_D_ I-domain binding to all previously identified ligands including fibrinogen, fibronectin, ICAM-3, VCAM-1, plasminogen, vitronectin, and CYR61 using the surface plasmon resonance (Biacore 3000) (11). These studies demonstrated a K_D_ for α_D_ I-domain/fibrinogen binding 7.6x10^-7^ M. In another project we tested α_D_ I-domain binding to CEP by Biacore and calculated constant dissociation for this binding as K_D_ 1.8x10^-7^ M (23). However, we did not compare α_D_ I-domain binding to fibrinogen and CEP in the same experiment. Previously, the Biacore results for α_D_ binding to fibrinogen and to CEP were obtained from different α_D_ I-domain isolations, using different ligand densities on sensor chips and were performed several years apart (11, 23). Therefore, it was critical to analyze these interactions in one experiment to verify this result. Based on Biolayer Interfereometry results, which were performed on one α_D_ I-domain isolation using the same I-domain concentrations, we found that the calculated affinity of α_D_ I-domain/CEP interaction (1.0x10^-7^M) is 2 folds stronger compared to the α_D_ I-domain/fibrinogen binding (2.2x10^-7^M). These results are slightly different from our previous Biocare results. We explain this difference by the difference in the experimental platforms between surface plasmon resonance (Biocore) and biolayer interferometry (Octet). Nevertheless, both methods provides a similar result and demonstrates the stronger binding of α_D_ I-domain to CEP compared to fibrinogen.

All α_D_ ligands analyzed by Biacore previously (11) showed a K_D_ for α_D_ I-domain binding in the range 0.2-8x10^-6^ M. The strongest binding was found for α_D_ I-domain interaction with the rare ECM protein CYR61 (CCN1) with the K_D_ 2.9x10^-7^ M. This affinity is still weaker than α_D_ I-domain/CEP binding that we detected. Most importantly, the binding to “classical” α_D_β_2_ ligands such as ICAM-3 (1.89x10^-6^ M), VCAM-1 (8.13x10^-6^ M), and fibrinogen (0.76x10^-6^ M) (11) are significantly less compared to CEP. These data together demonstrate that CEP modification generates preferential ligands for α_D_β_2_-mediated macrophage adhesion during inflammation. Therefore, inflammation and oxidation generate the improved conditions for α_D_β_2_-dependent macrophage adhesion.

The theory of mesenchymal cell migration postulates that optimal migration occurs with an intermediate level of receptor expression and substrate density, while the high density of receptors and corresponding substrates generate very strong adhesion, preventing cell motility (7, 26, 44–46).

Previously we found that non-activated macrophages demonstrated an improved migration in a CEP-supplemented matrix (23). Non-activated macrophages express an intermediate level of α_D_β_2_ and α_M_β_2_ integrins (26). We and others have shown experimentally that intermediate levels of integrin expression support cell migration (7, 26, 47, 48).

We propose that upregulation of α_D_β_2_ on M1 macrophages and modification of ECM proteins with CEP promotes the conditions for the formation of an excessive number of receptor-ligand pairs with a high binding affinity. Accordingly, this high level of macrophage adhesion can prevent macrophage migration. In agreement with this concept, we found that migration of M1 macrophages in a 3D fibrin matrix supplemented with CEP is significantly reduced compared to migration in a pure fibrin matrix. Interestingly, we found that the morphology of M1 macrophages in a 3D matrix is affected by the presence of CEP (**Figure 4D**). Particularly, we revealed, that M1 macrophages in CEP supplemented matrix have a more rounded shape compared to macrophages in fibrin gel. It has been shown previously that migrated macrophages have an elongated shape and accordingly a greater length-to-diameter ratio (49, 50). The length-to-diameter ratio of macrophages in a CEP supplemented matrix is statistically significantly lower compared to control, which is in agreement with the reduced macrophage migration that we detected (**Figure 4A**).

To summarize, we propose a potential mechanism that affects macrophage adhesion and migration in inflamed tissue. Oxidation of DHA, which is a common structural component of cell membranes, leads to the formation of adducts between ECM proteins and CEP. Experimental data demonstrated that 20-30 modifications can occur in one protein molecule (23, 30, 32, 51). These modifications substitute a lysine-mediated positive charge for a CEP-mediated negative charge, which affects the conformation and ligand-binding properties of modified proteins. CEP is recognized by integrins α_M_β_2_ and α_D_β_2_. Accordingly, CEP modification can transform random proteins in integrin-related ligands. Freshly differentiated non-activated macrophages possess an intermediate level of α_M_β_2_ and α_D_β_2_ expression that supports CEP-mediated migration to the site of inflammation. Polarized pro-inflammatory (M1-like) macrophages express a high level of α_D_β_2,_ generating a high adhesion to CEP-modified proteins in the inflamed tissue that prevents M1 macrophage migration and leads to the development of chronic inflammation. The high affinity between α_D_β_2_ and CEP additionally intensifies the potential effect. This model proposes that targeting the binding between α_D_β_2_ and CEP can provide benefits against macrophage retention in the inflamed tissue and the development of chronic inflammation during metabolic and autoimmune diseases.

## Data Availability Statement

The original contributions presented in the study are included in the article/supplementary material. Further inquiries can be directed to the corresponding author.

## Ethics Statement

The animal study was reviewed and approved by East Tennessee State University IACUC.

## Author Contributions

JC performed the experiments, analyzed the data, and edited the manuscript; KK, DG, CA, performed the experiments and analyzed the data; DW, EP, TB analyzed the data and edited the manuscript; VY designed the research, performed the experiments, analyzed the data, and wrote the manuscript. All authors contributed to the article and approved the submitted version.

## Funding

These studies were supported by the American Heart Association 20AIREA35150018 (VY); and partially supported by the National Institutes of Health grants R15HL157836 (VY), R01GM119197 (DW), R01GM083016 (DW), R01HL071625 (TB), R01HL145536 (TB) and by the National Institute of Health grant C06RR0306551 for East Tennessee State University.

## Conflict of Interest

The authors declare that the research was conducted in the absence of any commercial or financial relationships that could be construed as a potential conflict of interest.

## Publisher’s Note

All claims expressed in this article are solely those of the authors and do not necessarily represent those of their affiliated organizations, or those of the publisher, the editors and the reviewers. Any product that may be evaluated in this article, or claim that may be made by its manufacturer, is not guaranteed or endorsed by the publisher.

## Abbreviations

CEP, carboxyethylpyrrole; ECM, Extracellular matrix; EDTA, Ethylenediaminetetraacetic acid; FACS, Fluorescence-activated cell sorting; IFNγ, interferon-γ; CCL2, C-C motif chemokine ligand 2 (MCP-1); CCL5, C-C motif chemokine ligand 5 (RANTES); TG, thioglycollate; WT, wide type; 2D, 2 dimensional; 3D, 3 dimensional.

## References

[1] Parisi L Gini E Baci D Tremolati M Fanuli M Bassani B. Macrophage Polarization in Chronic Inflammatory Diseases: Killers or Builders? J Immunol Res (2018) 2018:8917804. doi: 10.1155/2018/8917804 29507865 PMC5821995

[2] Groh L Keating ST Joosten LAB Netea MG Riksen NP. Monocyte and Macrophage Immunometabolism in Atherosclerosis. Semin Immunopathol (2018) 40(2):203–14. doi: 10.1007/s00281-017-0656-7 PMC5809534 28971272

[3] Ross EA Devitt A Johnson JR. Macrophages: The Good, the Bad, and the Gluttony. Front Immunol (2021) 12:708186. doi: 10.3389/fimmu.2021.708186 34456917 PMC8397413

[4] Koltsova EK Hedrick CC Ley K. Myeloid Cells in Atherosclerosis: A Delicate Balance of Anti-Inflammatory and Proinflammatory Mechanisms. Curr Opin Lipidol (2013) 24(5):371–80. doi: 10.1097/MOL.0b013e328363d298 PMC4939820 24005215

[5] Locati M Curtale G Mantovani A. Diversity, Mechanisms, and Significance of Macrophage Plasticity. Annu Rev Pathol (2020) 15:123–47. doi: 10.1146/annurev-pathmechdis-012418-012718 PMC7176483 31530089

[6] Moore KJ Sheedy FJ Fisher EA. Macrophages in Atherosclerosis: A Dynamic Balance. Nat Rev Immunol (2013) 13(10):709–21. doi: 10.1038/nri3520 PMC4357520 23995626

[7] Palecek SP Loftus JC Ginsberg MH Lauffenburger DA Horwitz AF. Integrin-Ligand Binding Properties Govern Cell Migration Speed Through Cell-Substratum Adhesiveness. Nature (1997) 385:537–40. doi: 10.1038/385537a0 9020360

[8] Ammon C Meyer SP Schwarzfischer L Krause SW Andreesen R Kreutz M. Comparative Analysis of Integrin Expression on Monocyte-Derived Macrophages and Monocyte-Derived Dendritic Cells. Immunology (2000) 100(3):364–9. doi: 10.1046/j.1365-2567.2000.00056.x PMC2327027 10929059

[9] Ou Z Dolmatova E Lassegue B Griendling KK. β1- and β2-Integrins: Central Players in Regulating Vascular Permeability and Leukocyte Recruitment During Acute Inflammation. Am J Physiol Heart Circ Physiol (2021) 320(2):H734–H9. doi: 10.1152/ajpheart.00518.2020 PMC8082787 33337960

[10] Yakubenko VP Lishko VK Lam SC-T Ugarova TP. A Molecular Basis for Integrin α_M_β_2_ in Ligand Binding Promiscuity. J Biol Chem (2002) 277:48635–42. doi: 10.1074/jbc.M208877200 12377763

[11] Yakubenko VP Yadav SP Ugarova TP. Integrin α_D_β_2_, an Adhesion Receptor Up-Regulated on Macrophage Foam Cells, Exhibits Multiligand-Binding Properties. Blood (2006) 107:1643–50. doi: 10.1182/blood-2005-06-2509 PMC1367263 16239428

[12] Aziz MH Cui K Das M Brown KE Ardell CL Febbraio M. The Upregulation of Integrin α_D_β_2_ (CD11d/CD18) on Inflammatory Macrophages Promotes Macrophage Retention in Vascular Lesions and Development of Atherosclerosis. J Immunol (2017) 198(12):4855–67. doi: 10.4049/jimmunol.1602175 PMC5553324 28500072

[13] Danilenko DM Rossito PV van der Vieren M Le Tring H Mc Donagh SP Affolter VK. A Novel Canine Leukointegrin, Alpha D Beta 2, is Expressed by Specific Macrophage Subpopulations in Tissue and a Minot CD8+ Lymphocyte Subpopulation in Peripheral Blood. J Immunol (1995) 155:35–44. 7541420

[14] Van der Vieren M Le Trong H St.John T Staunton DE Gallatin WM. A Novel Leukointegrin, α_D_β_2_, Binds Preferentially to ICAM-3. Immunity (1995) 3:683–90. doi: 10.1016/1074-7613(95)90058-6 8777714

[15] Bao F Brown A Dekaban GA Omana V Weaver LC. CD11d Integrin Blockade Reduces the Systemic Inflammatory Response Syndrome After Spinal Cord Injury. Exp Neurol (2011) 231(2):272–83. doi: 10.1016/j.expneurol.2011.07.001 PMC4853193 21784069

[16] Thomas AP Dunn TN Oort PJ Grino M Adams SH. Inflammatory Phenotyping Identifies CD11d as a Gene Markedly Induced in White Adipose Tissue in Obese Rodents and Women. J Nutr (2011) 141(6):1172–80. doi: 10.3945/jn.110.127068 21508205

[17] Weaver LC Bao F Dekaban GA Hryciw T Shultz SR Cain DP. CD11d Integrin Blockade Reduces the Systemic Inflammatory Response Syndrome After Traumatic Brain Injury in Rats. Exp Neurol (2015) 271:409–22. doi: 10.1016/j.expneurol.2015.07.003 PMC4854624 26169930

[18] Miyazaki Y Bunting M Stafforini DM Harris ES McIntyre TM Prescott SM. Integrin α_D_β_2_ Is Dynamically Expressed by Inflamed Macrophages and Alters the Natural History of Lethal Systemic Infections. J Immunol (2008) 180(1):590–600. doi: 10.4049/jimmunol.180.1.590 18097061 PMC2275910

[19] de Azevedo-Quintanilha IG Vieira-de-Abreu A Ferreira AC Nascimento DO Siqueira AM Campbell RA. Integrin α_D_β_2_ (CD11d/CD18) Mediates Experimental Malaria-Associated Acute Respiratory Distress Syndrome (MA-ARDS). Malar J (2016) 15(1):393. doi: 10.1186/s12936-016-1447-7 27473068 PMC4967320

[20] Nascimento DO Vieira-de-Abreu A Arcanjo AF Bozza PT Zimmerman GA Castro-Faria-Neto HC. Integrin α_D_β_2_ (CD11d/CD18) Modulates Leukocyte Accumulation, Pathogen Clearance, and Pyroptosis in Experimental Salmonella Typhimurium Infection. Front Immunol (2018) 9:1128. doi: 10.3389/fimmu.2018.01128 29881383 PMC5977906

[21] Bailey WP Cui K Ardell CL Keever KR Singh S Rodriguez-Gil DJ. The Expression of Integrin α_D_β_2_ (CD11d/CD18) on Neutrophils Orchestrates the Defense Mechanism Against Endotoxemia and Sepsis. J Leukoc Biol (2021) 109(5):877–90. doi: 10.1002/JLB.3HI0820-529RR. PMC8085079 33438263

[22] Yakubenko VP Byzova TV. Biological and Pathophysiological Roles of End-Products of DHA Oxidation. Biochim Biophys Acta (2017) 1862(4):407–15. doi: 10.1016/j.bbalip.2016.09.022 PMC5360178 27713004

[23] Yakubenko VP Cui K Ardell CL Brown KE West XZ Gao D. Oxidative Modifications of Extracellular Matrix Promote the Second Wave of Inflammation *via* β_2_ Integrins. Blood (2018) 132(1):78–88. doi: 10.1182/blood-2017-10-810176 29724896 PMC6034644

[24] Blythe EN Weaver LC Brown A Dekaban GA. Beta2 Integrin CD11d/CD18: From Expression to an Emerging Role in Staged Leukocyte Migration. Front Immunol (2021) 12:775447. doi: 10.3389/fimmu.2021.775447 34858434 PMC8630586

[25] Shi C Simon DI. Integrin Signals, Transcription Factors, and Monocyte Differentiation. Trends Cardiovasc Med (2006) 16(5):146–52. doi: 10.1016/j.tcm.2006.03.002 16781947

[26] Cui K Ardell CL Podolnikova NP Yakubenko VP. Distinct Migratory Properties of M1, M2, and Resident Macrophages Are Regulated by α_D_β_2_ and α_M_β_2_ Integrin-Mediated Adhesion. Front Immunol (2018) 9:2650. doi: 10.3389/fimmu.2018.02650 30524429 PMC6262406

[27] Szpak D Izem L Verbovetskiy D Soloviev DA Yakubenko VP Pluskota E. Alphambeta2 Is Antiatherogenic in Female But Not Male Mice. J Immunol (2018) 200(7):2426–38. doi: 10.4049/jimmunol.1700313 PMC5973813 29459405

[28] Wolf D Bukosza N Engel D Poggi M Jehle F Anto MN. Inflammation, But Not Recruitment, of Adipose Tissue Macrophages Requires Signalling Through Mac-1 (CD11b/CD18) in Diet-Induced Obesity (DIO). Thromb Haemost (2017) 117(2):325–38. doi: 10.1160/TH16-07-0553 27853810

[29] Miyazaki Y Vieira-de-Abreu A Harris ES Shah AM Weyrich AS Castro-Faria-Neto HC. Integrin α_D_β_2_ (CD11d/CD18) Is Expressed by Human Circulating and Tissue Myeloid Leukocytes and Mediates Inflammatory Signaling. PloS One (2014) 9(11):e112770. doi: 10.1371/journal.pone.0112770 25415295 PMC4240710

[30] Gu X Meer SG Miyagi M Rayborn ME Hollyfield JG Crabb JW. Carboxyethylpyrrole Protein Adducts and Autoantibodies, Biomarkers for Age-Related Macular Degeneration. J Biol Chem (2003) 278(43):42027–35. doi: 10.1074/jbc.M305460200 12923198

[31] West XZ Malinin NL Merkulova AA Tischenko M Kerr BA Borden EC. Oxidative Stress Induces Angiogenesis by Activating TLR2 With Novel Endogenous Ligands. Nature (2010) 467(7318):972–6. doi: 10.1038/nature09421 PMC2990914 20927103

[32] Ebrahem Q Renganathan K Sears J Vasanji A Gu X Lu L. Carboxyethylpyrrole Oxidative Protein Modifications Stimulate Neovascularization: Implications for Age-Related Macular Degeneration. Proc Natl Acad Sci USA (2006) 103(36):13480–4. doi: 10.1073/pnas.0601552103 PMC1569188 16938854

[33] Hoff HF O'Neil J Wu Z Hoppe G Salomon RL. Phospholipid Hydroxyalkenals: Biological and Chemical Properties of Specific Oxidized Lipids Present in Atherosclerotic Lesions. Arterioscler Thromb Vasc Biol (2003) 23(2):275–82. doi: 10.1161/01.ATV.0000051407.42536.73 12588771

[34] Kim YW Yakubenko VP West XZ Gugiu GB Renganathan K Biswas S. Receptor-Mediated Mechanism Controlling Tissue Levels of Bioactive Lipid Oxidation Products. Circ Res (2015) 117(4):321–32. doi: 10.1161/CIRCRESAHA.117.305925 PMC4522201 25966710

[35] Wolf D Anto-Michel N Blankenbach H Wiedemann A Buscher K Hohmann JD. A Ligand-Specific Blockade of the Integrin Mac-1 Selectively Targets Pathologic Inflammation While Maintaining Protective Host-Defense. Nat Commun (2018) 9(1):525. doi: 10.1038/s41467-018-02896-8 29410422 PMC5802769

[36] Lishko VK Burke T Ugarova T. Antiadhesive Effect of Fibrinogen: A Safeguard for Thrombus Stability. Blood (2007) 109(4):1541–9. doi: 10.1182/blood-2006-05-022764 PMC1794063 16849640

[37] Yermolenko IS Gorkun OV Fuhrmann A Podolnikova NP Lishko VK Oshkadyerov SP. The Assembly of Nonadhesive Fibrinogen Matrices Depends on the alphaC Regions of the Fibrinogen Molecule. J Biol Chem (2012) 287(50):41979–90. doi: 10.1074/jbc.M112.410696 PMC3516744 23086938

[38] Kerr BA Ma L West XZ Ding L Malinin NL Weber ME. Interference With Akt Signaling Protects Against Myocardial Infarction and Death by Limiting the Consequences of Oxidative Stress. Sci Signal (2013) 6(287):ra67. doi: 10.1126/scisignal.2003948 23921086 PMC3971949

[39] Hollyfield JG Bonilha VL Rayborn ME Yang X Shadrach KG Lu L. Oxidative Damage-Induced Inflammation Initiates Age-Related Macular Degeneration. Nat Med (2008) 14(2):194–8. doi: 10.1038/nm1709 PMC2748836 18223656

[40] Shoulders MD Raines RT. Collagen Structure and Stability. Annu Rev Biochem (2009) 78:929–58. doi: 10.1146/annurev.biochem.77.032207.120833 PMC2846778 19344236

[41] Cui K Podolnikova NP Bailey W Szmuc E Podrez EA Byzova TV. Inhibition of Integrin α_D_β_2-_Mediated Macrophage Adhesion to End-Product of DHA Oxidation Prevents Macrophage Accumulation During Inflammation. J Biol Chem (2019) 294(39):14370–82. doi: 10.1074/jbc.RA119.009590 PMC6768641 31395659

[42] Vorup-Jensen T Carman CV Shimaoka M Schuck P Svitel J Springer TA. Exposure of Acidic Residues as a Danger Signal for Recognition of Fibrinogen and Other Macromolecules by Integrin α_X_β_2_ . Proc Natl Acad Sci USA (2005) 102(5):1614–9. doi: 10.1073/pnas.0409057102 PMC547869 15665082

[43] Luo BH Carman CV Springer TA. Structural Basis of Integrin Regulation and Signaling. Annu Rev Immunol (2007) 25:619–47. doi: 10.1146/annurev.immunol.25.022106.141618 PMC1952532 17201681

[44] Wiesner C Le-Cabec V El AK Maridonneau-Parini I Linder S. Podosomes in Space: Macrophage Migration and Matrix Degradation in 2D and 3D Settings. Cell Adh Migr (2014) 8(3):179–91. doi: 10.4161/cam.28116 PMC4198342 24713854

[45] DiMilla PA Barbee K Lauffenburger DA. Mathematical Model for the Effects of Adhesion and Mechanics on Cell Migration Speed. Biophys J (1991) 60:15–37. doi: 10.1016/S0006-3495(91)82027-6 1883934 PMC1260035

[46] DiMilla PA Stone JA Quinn JA Albelda SM Lauffenburger DA. Maximal Migration of Human Smooth Muscle Cells on Fibronectin and Type IV Collagen Occurs at an Intermediate Attachment Strength. J Cell Biol (1993) 122:729–37. doi: 10.1083/jcb.122.3.729 PMC2119669 8335696

[47] Lishko VK Yakubenko VP Ugarova TP. The Interplay Between Integrins α_M_β_2_ and α_5_β_1_ During Cell Migration to Fibronectin. Exp Cell Res (2003) 283:116–26. doi: 10.1016/S0014-4827(02)00024-1 12565824

[48] Yakubenko VP Belevych N Mishchuk D Schurin A Lam SC Ugarova TP. The Role of Integrin Alpha D Beta2 (CD11d/CD18) in Monocyte/Macrophage Migration. Exp Cell Res (2008) 314:2569–78. doi: 10.1016/j.yexcr.2008.05.016 PMC2621015 18621369

[49] Cougoule C Van GE Le C Lafouresse F Dupre L Mehraj V. Blood Leukocytes and Macrophages of Various Phenotypes Have Distinct Abilities to Form Podosomes and to Migrate in 3D Environments. Eur J Cell Biol (2012) 91(11-12):938–49. doi: 10.1016/j.ejcb.2012.07.002 22999511

[50] McWhorter FY Wang T Nguyen P Chung T Liu WF. Modulation of Macrophage Phenotype by Cell Shape. Proc Natl Acad Sci USA (2013) 110(43):17253–8. doi: 10.1073/pnas.1308887110 PMC3808615 24101477

[51] Salomon RG. Carboxyethylpyrroles: From Hypothesis to the Discovery of Biologically Active Natural Products. Chem Res Toxicol (2017) 30(1):105–13. doi: 10.1021/acs.chemrestox.6b00304 PMC5382959 27750413

